# The *Caenorhabditis elegans* Gene *mfap-1* Encodes a Nuclear Protein That Affects Alternative Splicing

**DOI:** 10.1371/journal.pgen.1002827

**Published:** 2012-07-19

**Authors:** Long Ma, Xiaoyang Gao, Jintao Luo, Liange Huang, Yanling Teng, H. Robert Horvitz

**Affiliations:** 1 State Key Laboratory of Medical Genetics, School of Biological Sciences and Technology, Central South University, Changsha, China; 2 Howard Hughes Medical Institute, Department of Biology, Massachusetts Institute of Technology, Cambridge, Massachusetts, United States of America; University of California San Diego, United States of America

## Abstract

RNA splicing is a major regulatory mechanism for controlling eukaryotic gene expression. By generating various splice isoforms from a single pre–mRNA, alternative splicing plays a key role in promoting the evolving complexity of metazoans. Numerous splicing factors have been identified. However, the *in vivo* functions of many splicing factors remain to be understood. *In vivo* studies are essential for understanding the molecular mechanisms of RNA splicing and the biology of numerous RNA splicing-related diseases. We previously isolated a *Caenorhabditis elegans* mutant defective in an essential gene from a genetic screen for suppressors of the rubberband Unc phenotype of *unc-93(e1500)* animals. This mutant contains missense mutations in two adjacent codons of the *C. elegans* microfibrillar-associated protein 1 gene *mfap-1*. *mfap-1(n4564 n5214)* suppresses the Unc phenotypes of different rubberband Unc mutants in a pattern similar to that of mutations in the splicing factor genes *uaf-1* (the *C. elegans* U2AF large subunit gene) and *sfa-1* (the *C. elegans* SF1/BBP gene). We used the endogenous gene *tos-1* as a reporter for splicing and detected increased intron 1 retention and exon 3 skipping of *tos-1* transcripts in *mfap-1(n4564 n5214)* animals. Using a yeast two-hybrid screen, we isolated splicing factors as potential MFAP-1 interactors. Our studies indicate that *C. elegans mfap-1* encodes a splicing factor that can affect alternative splicing.

## Author Summary

RNA splicing removes intervening intronic sequences from pre–mRNA transcripts and joins adjacent exonic sequences to generate functional messenger RNAs. The *in vivo* functions of numerous factors that regulate splicing remain to be understood. From a genetic screen for suppressors of the rubberband Unc phenotype caused by the *Caenorhabditis elegans unc-93(e1500)* mutation, we isolated a mutation that affects a highly conserved essential gene, *mfap-1*. MFAP-1 is a nuclear protein that is broadly expressed. MFAP-1 can affect the alternative splicing of *tos-1*, an endogenous reporter gene for splicing, and is required for the altered splicing at a cryptic 3′ splice site of *tos-1*. *mfap-1* enhances the effects of the gene *uaf-1* (splicing factor U2AF large subunit) in suppressing the rubberband Unc phenotype of *unc-93(e1500)* animals. Our studies provide *in vivo* evidence that MFAP-1 functions as a splicing factor.

## Introduction

RNA splicing removes non-coding introns and joins adjacent coding exons from pre-mRNAs to generate functional coding mRNAs. Alternative splicing can generate numerous splice isoforms from the same pre-mRNA [1]. The complex proteome encoded by mRNA splice isoforms is believed to be a major driving force for the evolving complexity of metazoans [1], [2]. Numerous proteins and non-coding RNAs regulate RNA splicing [3]. The U1 snRNP complex and the SF1/U2AF65/U2AF35 protein complex recognize the 5′ and 3′ splice sites of an intron, respectively [4]–[11], and the U2 and U4/U5/U6 snRNP complexes assemble in a step-wise manner and undergo compositional and conformational rearrangements to drive the two steps of the trans-esterification reaction in RNA splicing [7], [12]. Mutations in *trans*-splicing factors or *cis*-regulatory splicing elements cause numerous diseases [13], [14].

Over 200 protein factors have been shown to regulate splicing or associate with the splicing machinery or other splicing factors [3]. Splicing factors have been identified mostly using biochemical approaches, and the *in vivo* functions of many splicing factors remain largely unknown. *In vivo* analysis of these factors remains a challenge, since many are essential for viability and the analyses of their *in vivo* functions can be limited by the lethality caused by mutations in these factors.

Recent studies indicate that conclusions concerning splicing factors derived from *in vitro* analyses should be complemented by *in vivo* analyses. For example, *in vivo* studies suggest that the splicing factor SF1/BBP, once thought to be ubiquitously required for splicing, might be required for the splicing of only a subset of genes [15]–[17]. Some *Saccharomyces cerevisiae* core splicing factors, once thought to be essential for the splicing of all introns, were found to affect splicing of only a subgroup of introns when the effects of loss-of-function mutations in these factors were examined [18]. Also, the functions of some splicing factors extend beyond regulating RNA splicing. For example, the U2AF large subunit is required for the efficient export of intronless mRNAs in *Drosophila* in addition to its essential role in regulating 3′ splice site recognition [19]. Thus, *in vivo* functional analyses are essential for an accurate understanding of the biological functions of splicing factors.

In *Caenorhabditis elegans*, the genes *unc-93*, *sup-9* and *sup-10* encode components of a presumptive two-pore domain K+ channel complex that affects muscle activity [20]–[23]. Animals carrying rare gain-of-function (gf) mutations in any of these three genes are sluggish, defective in egg laying and exhibit a rubberband phenotype: when touched on the head, the animal contracts and relaxes along its entire body without moving backwards. Complete loss-of-function (lf) mutations of *unc-93, sup-9* or *sup-10* do not cause obvious abnormalities [21], [22]. The SUP-9 protein is similar to the mammalian Two-pore Acid Sensitive K+ channels TASK-1 and TASK-3 [20]. *sup-10* encodes a novel single-transmembrane domain protein without identified mammalian homologs [20], and *unc-93* encodes a multiple transmembrane-domain protein that defines a novel family of proteins conserved from *C. elegans* to mammals [20], [23]. A mammalian UNC-93 homolog, UNC-93b, interacts with Toll-like receptors and regulates innate immune responses [24]–[28].

We previously screened for new suppressors of the “rubberband” Unc phenotype of *unc-93(e1500)* animals and isolated mutations affecting the splicing factors U2AF large subunit (UAF-1) and SF1/BBP (SFA-1) [29]. Our analysis suggested that mutations in *uaf-1* and *sfa-1* result in the suppression of the rubberband Unc phenotype of *unc-93(e1500)* animals by altering the splicing of the pre-mRNA of an unknown gene [29]. We identified the pre-mRNA of the gene *tos-1* as abnormally spliced in *uaf-1* and *sfa-1* mutants and determined that *tos-1* is a sensitive endogenous reporter for analyzing *in vivo* functions of splicing factors [30]. In this study, we describe a third isolate, *n4564 n5214*, from the genetic screen in which we identified the *uaf-1* and *sfa-1* mutations [29]. We found that the gene affected by *n4564 n5214* encodes a novel splicing factor that affects alternative splicing in *C. elegans*.

## Results

### *n4564 n5214* suppresses the rubberband Unc phenotype of *unc-93(e1500)* animals

Previously we performed a genetic screen for mutations that caused sterility and/or lethality and concurrently suppressed the rubberband Unc phenotype caused by the *unc-93(e1500)* mutation [29]. Besides *uaf-1(n4588)* and *sfa-1(n4562)*, we also isolated the mutation *n4564* [29], which we renamed *n4564 n5214* in this study (see below). Although the precise mechanisms underlying the suppression of the rubberband Unc phenotypes by the *uaf-1, sfa-1* [29] and *n4564 n5214* (see below) mutations remain to be determined, the *uaf-1* and *sfa-1* mutations we isolated and the splicing of the *uaf-1* target *tos-1* can provide tools for studying *in vivo* functions of splicing factor genes [29], [30], which we now report include the gene affected by *n4564 n5214*.

*n4564 n5214* mutants exhibited temperature-sensitive lethality: at 15°C, *n4564 n5214* homozygous animals grew and behaved similarly to the wild type; at 20°C, mutant animals grew more slowly, had few progeny and were hyperactive (Table 1); at 25°C, the mutant strain was embryonically lethal. *n4564 n5214* weakly suppressed the Unc phenotype of *unc-93(e1500)* animals at 15°C (L. Ma and H. R. Horvitz, unpublished observations) and was a stronger suppressor at 20°C (Table 1). *n4564 n5214/+; unc-93(e1500)* animals were as Unc as *unc-93(e1500)* animals (L. Ma and H. R. Horvitz, unpublished observations), indicating that *n4564 n5214* is a recessive suppressor of the Unc phenotype of *unc-93(e1500)* animals and suggesting that *n4564 n5214* causes a reduction or loss of *mfap-1* function. We tested whether *n4564 n5214* also suppressed the Unc phenotype caused by the rubberband mutants *unc-93(n200)*, *sup-9(n1550)* and *sup-10(n983)*. *n200* is a weakly semi-dominant *unc-93* allele that causes a weak rubberband Unc phenotype [22]. The *sup-9(n1550)* and *sup-10(n983)* gain-of-function mutations cause strong and moderate rubberband Unc phenotypes, respectively [20], [21], [23], [31]. *n4564 n5214* strongly suppressed *sup-10(n983)* but did not suppress *unc-93(n200)* or *sup-9(n1550)* (Table 1). Thus, *n4564 n5214* suppressed the same rubberband Unc mutants as do the *uaf-1(n4588)* and *sfa-1(n4562)* mutations, both of which suppress the rubberband Unc phenotypes of *unc-93(e1500)* and *sup-10(n983)* animals but not the rubberband Unc phenotypes of *unc-93(n200)* or *sup-9(n1550)* animals [29].

**Table 1:** Suppression of the rubberband Unc phenotype by *mfap-1* mutations.

Genotype	Bodybends per 30 sec (± SD)	n
*wild-type*	21.8±3.4	20
*wild-type* (25°C)	28.3±4.4	20
*unc-93(e1500)*	0.9±1.3	20
*unc-93(e1500)* (25°C)	0.8±0.8	20
*unc-93(n200)* [29]	15.6±3.5	20
*sup-10(n983)* [29]	4.3±1.9	20
*sup-9(n1550); sup-18(n1014)* [29]	0.1±0.4	20
*mfap-1(n4564 n5214)*	27.1±2.2	20
*mfap-1(n5214)*	28.4±3.3	20
*mfap-1(n5214)* (25°C)	28.3±4.0	20
*mfap-1(n4564 n5214); unc-93(e1500)*	21.8±4.5	20
*mfap-1(n4564 n5214); unc-93(n200)*	13.9±3.0	20
*mfap-1(n4564 n5214); sup-10(n983)*	18.0±4.1	20
*mfap-1(n4564 n5214); sup-9(n1550); sup-18(n1014)*	0.3±0.5	20
*mfap-1(n5214); unc-93(e1500)*	1.8±1.3	20
*mfap-1(n5214); unc-93(e1500)* (25°C)	8.0±3.6	20

### *n4564 n5214* contains two missense mutations in the gene *mfap-1,* which encodes a highly conserved nuclear protein

**Figure 1:**
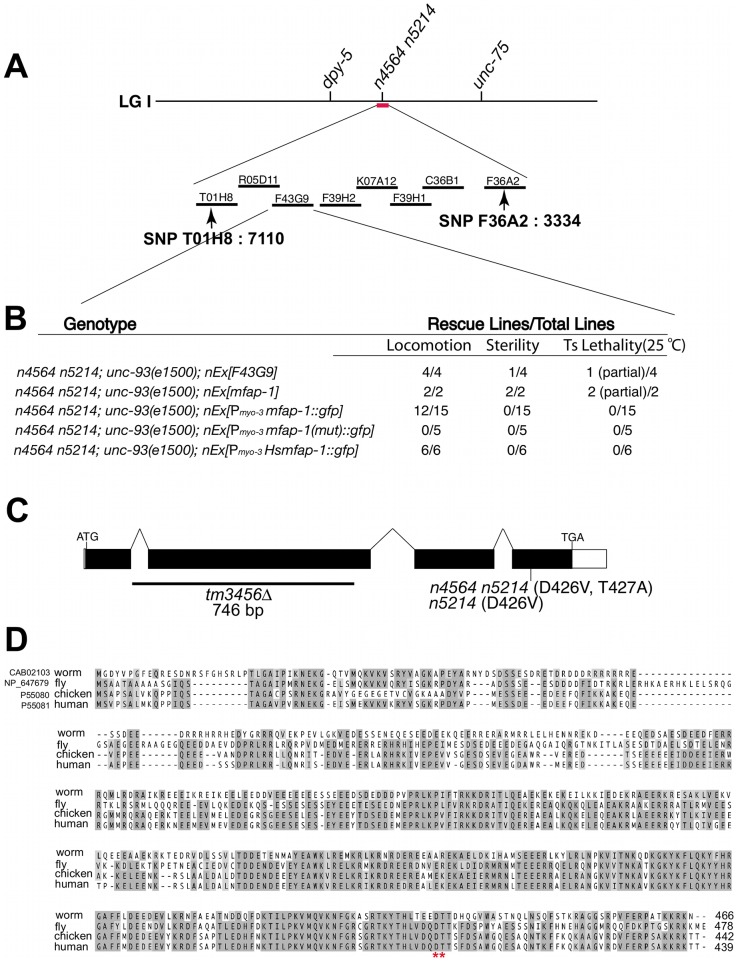
Genetic mapping, cloning, and identification of *mfap-1*. (A) Genomic location of the *n4564 n5214* mutation based on genetic mapping using visible markers and SNPs (*e.g.*, SNP T01H8: 7110 on the left and SNP F36A2: 3334 on the right). Cosmids tested in rescue experiments are labeled. Only cosmid *F43G9* rescued the three phenotypic characteristics of *mfap-(n4564 n5214)* individuals: suppression of the Unc phenotype of *unc-93(e1500)* animals; partial sterility at 20°C; and temperature-sensitive lethality at 25°C. (B) Transgene rescue experiments. Transgenes (*nEx*) were injected into the gonads of *mfap-1(n4564 n5214); unc-93(e1500)* animals, and lines stably transmitting the transgenes were established. Transgenic lines were analyzed for the rescue of each of three abnormalities: suppression of the Unc phenotype of *unc-93(e1500)* animals; partial sterility at 20°C; and temperature-sensitive lethality at 25°C. *mut*: the *n4564 n5214* mutation. (C) Gene structure of *mfap-1*. Black boxes: coding exons. Open boxes: 5′ and 3′ UTRs. Positions of start (ATG) and stop codons (TGA) are indicated. The sites of the *n4564 n5214* mutation, the *n5214* mutation and the *tm3456*Δ deletion are labeled. (D) Sequence alignment of predicted MFAP-1 proteins from *C. elegans*, *Drosophila*, chicken and human. *: amino acids mutated in *mfap-1(n4564 n5214)* mutants. Amino acids conserved in at least three orthologs are darkly shaded, while amino acids with similar physical properties or conserved in two orthologs are lightly shaded.

We cloned the gene affected by *n4564 n5214* by genetic mapping and transgene rescue experiments (Figure 1A, 1B; see Materials and Methods). The *n4564 n5214* strain contains two missense mutations in the gene *F43G9.10,* changing the adjacent conserved amino acids Asp 426 and Thr 427 to Val (*n5214*) and Ala (*n4564*) in the predicted protein, respectively (Figure 1C). *F43G9.10* encodes the *C. elegans* ortholog of a highly conserved mammalian protein that has been called “microfibrillar-associated protein 1” (41% identity between the *C. elegans* and human orthologs) (Figure 1D). We named *F43G9.10 mfap-1* (microfibrillar-associated protein 1). The chicken ortholog of MFAP-1 was suggested to be an extracellular matrix protein [32]. However, the *Drosophila* ortholog of MFAP-1 (dMFAP1) interacts with the splicing factor dPrp38, is required for normal expression of γ*-tubulin* and *stg/cdc25* mRNAs and has been proposed to act as a splicing factor [33]. We found that the expression of a human MFAP1::GFP fusion protein (*Hsmfap-1*) in *C. elegans* body-wall muscles rescued the suppression of *unc-93(e1500)* by *mfap-1(n4564 n5214)* (Figure 1B), indicating that the function of MFAP-1 is conserved from nematodes to humans.

**Figure 2:**
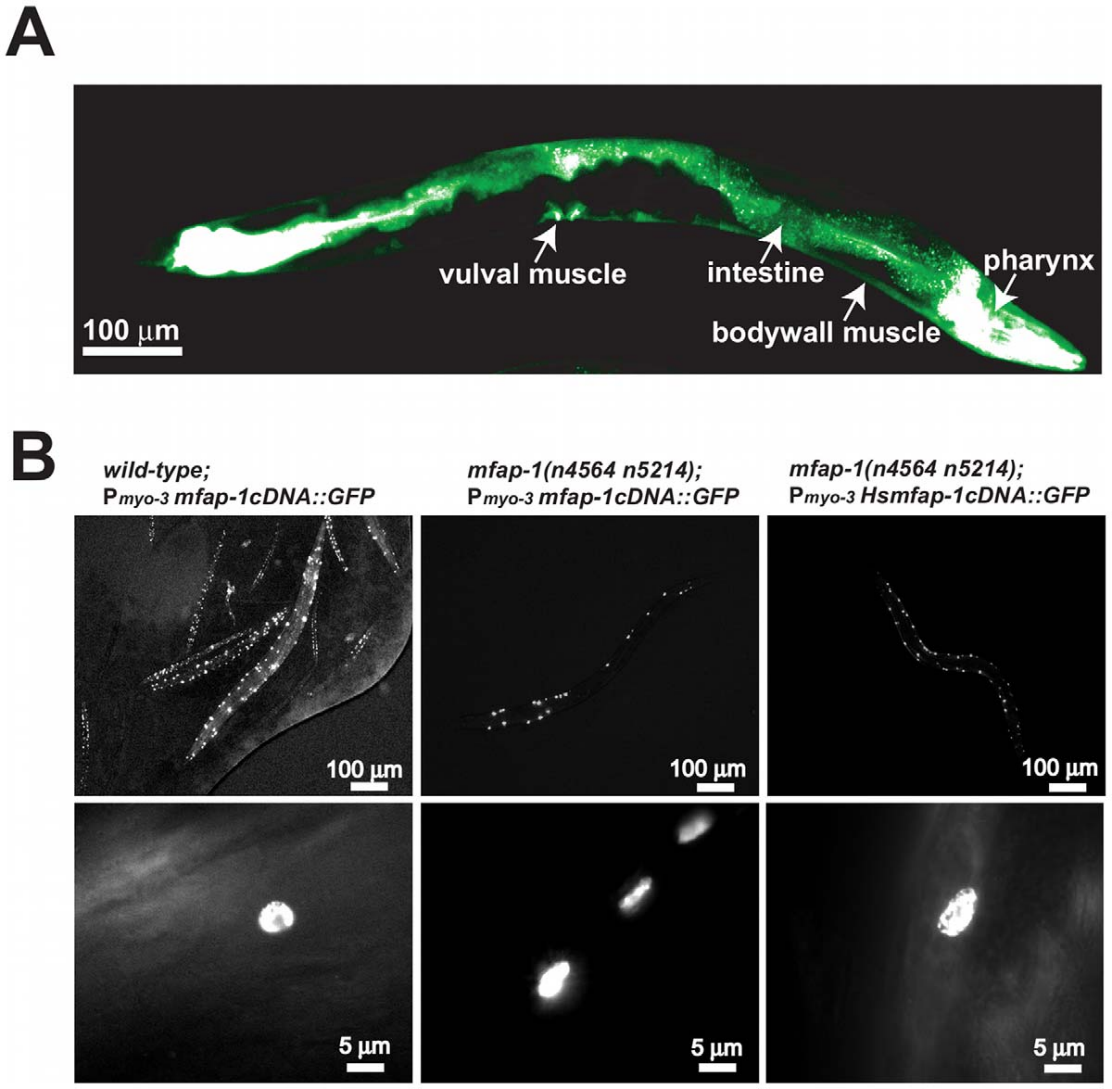
MFAP-1 is a nuclear protein expressed in multiple tissues. (A) A 2.5 kb 5′ promoter region upstream of the start codon of *mfap-1* was used to drive the expression of a transcriptional fusion GFP reporter in transgenic animals. The tissues in which the GFP reporter was expressed are labeled. This figure shows a montage of two photographs, which were pseudo-colored from a gray-scale image to a green-scale image using Adobe Photoshop to reflect the level of GFP expression. (B) Transgene experiments were performed as described in Materials and Methods. The *C. elegans* MFAP-1::GFP fusion protein, when expressed in body-wall muscles using a *myo-3* promoter in either wild-type or *mfap-1(n4564 n5214)* animals of mixed developmental stages, was exclusively localized in nuclei (left and middle panels). Similarly, human MFAP-1::GFP fusion protein, when expressed in the body-wall muscles of *mfap-1(n4564 n5214)* animals, was also exclusively localized in nuclei (right panels). Lower panels show typical nuclear GFP signals in adult transgenic animals. Genetic backgrounds and transgenes are indicated.

We examined the expression pattern of *mfap-1* in transgenic animals using a transcriptional fusion reporter construct that drives GFP expression under the control of an approximately 2.5 kb *mfap-1* promoter (Figure 2A). We observed strong GFP expression in the intestine, pharynx and vulval muscles (Figure 2A). We also observed GFP expression in the body-wall muscles (Figure 2A), consistent with the finding that body-wall muscle-specific expression of *mfap-1* rescued the suppression of the *unc-93(e1500)* Unc phenotype by *mfap-1(n4564 n5214)* (Figure 1). Both *C. elegans* MFAP-1::GFP and human MFAP-1::GFP, when expressed in body-wall muscles, were exclusively localized in nuclei (Figure 2B and unpublished observations), indicating that MFAP-1 is a nuclear protein.

We obtained a 746 bp *mfap-1* deletion allele, *tm3456*Δ (kindly provided by S. Mitani, personal communication), which removes the entire first intron and most of the second exon of *mfap-1* (Figure 1C). *tm3456*Δ is predicted to encode a truncated protein with a frameshift after amino acid 53, suggesting that *tm3456*Δ is likely a null allele of *mfap-1*. *mfap-1(tm3456*Δ)/+animals grew and behaved like the wild type, and *mfap-1(tm3456*Δ) homozygous animals arrested developmentally at the L1 or L2 larval stages. *tm3456*Δ/*n4564 n5214* animals similarly arrested at the L1/L2 larval stages, suggesting that the lethal phenotype of *tm3456*Δ homozygotes is caused by the *mfap-1(tm3456*Δ) mutation and consistent with the hypothesis that *mfap-1(n4564 n5214)* causes a reduction or loss of *mfap-1* function.

### The D426V(*n5214*) and T427A(*n4564*) mutations act together to suppress *unc-93(e1500)*

The temperature-sensitive lethal phenotype of *mfap-1(n4564 n5214)* at 25°C provided an approach to the identification of genes that interact with *mfap-1* by seeking suppressors of the temperature-sensitive lethality. We screened about 50,000 haploid genomes and identified two independent suppressors, which we later found to cause the same intragenic change in *mfap-1* (see Materials and Methods). Specifically, both suppressors caused a reversion of the T427A *(n4564)* mutation (see Materials and Methods) to the wild-type codon (ACA) and amino acid (A427T) and retained the D426V (*n5214*) mutation. *mfap-1(n5214)* did not obviously suppress the rubberband Unc phenotype of *unc-93(e1500)* animals at 20°C but did do so at 25°C (Table 1), indicating that *mfap-1(n5214)* is a temperature-sensitive allele.

We examined how the two *mfap-1* mutations, *n4564* and *n5214*, contribute to the activity of *mfap-1(n4564 n5214)* as a suppressor of the Unc phenotype of *unc-93(e1500)* animals. We expressed MFAP-1::GFP fusion transgenes containing the *n4564* (T427A) mutation, the *n5214* (D426V) mutation or both mutations in the body-wall muscles of *mfap-1(n4564 n5214); unc-93(e1500)* animals and assayed the locomotion of transgenic animals (Table 2). Both the *mfap-1(n4564)::GFP* and the *mfap-1(n5214)::GFP* transgenes greatly rescued the suppression of *unc-93(e1500)* by *mfap-1(n4564 n5214)*, while the *mfap-1(n4564 n5214)::GFP* transgene only partially rescued the suppression (Table 2). These results suggest that individually the mutations *n4564* or *n5214* only slightly if at all affect the function of *mfap-1* and that the strong suppression of *unc-93(e1500)* by *mfap-1(n4564 n5214)* probably results from an additive or synergistic effect of these two mutations, likely in reducing *mfap-1* function.

**Table 2:** *mfap-1(n4564)* and *mfap-1(n5214)* are weak mutations that act together to suppress the rubberband Unc phenotype of *unc-93(e1500)* animals.

Genotype	Bodybends per 30 sec (±SD)	n
*nEx[P_myo-3_ mfap-1 cDNA (wt)::GFP]* line 1	1.3±1.4	20
*nEx[P_myo-3_ mfap-1 cDNA (wt)::GFP]* line 2	2.1±2.1	20
*nEx[P_myo-3_ mfap-1 cDNA (wt)::GFP]* line 3	1.7±2.0	20
*nEx[P_myo-3_ mfap-1 cDNA (n4564)::GFP]* line 1	1.3±1.1	20
*nEx[P_myo-3_ mfap-1 cDNA (n4564)::GFP]* line 2	1.6±1.7	20
*nEx[P_myo-3_ mfap-1 cDNA (n4564)::GFP]* line 3	3.8±1.4	20
*nEx[P_myo-3_ mfap-1 cDNA (n5214)::GFP]* line 1	3.4±1.6	20
*nEx[P_myo-3_ mfap-1 cDNA (n5214)::GFP]* line 2	2.2±2.5	9
*nEx[P_myo-3_ mfap-1 cDNA (n5214)::GFP]* line 3	3.1±1.6	20
*nEx[P_myo-3_ mfap-1 cDNA (n4564 n5214)::GFP]* line 1	10.1±4.7	20
*nEx[P_myo-3_ mfap-1 cDNA (n4564 n5214)::GFP]* line 2	14.3±6.4	20

All transgenes were expressed in *mfap-1(n4564 n5214); unc-93(e1500)* animals. Two lines were obtained and analyzed for transgene *P_myo-3_ mfap-1 cDNA (n4564 n5214)::GFP*.

### *mfap-1* and *uaf-1* interact to affect the locomotion of *unc-93(e1500)* animals

To test for an interaction between *mfap-1* and *uaf-1*, we generated *mfap-1(n4564 n5214); uaf-1* mutant animals carrying the *unc-93(e1500)* mutation. Like *mfap-1(n4564 n5214)* animals, *mfap-1(n4564 n5214); uaf-1(n5123)* animals were viable at 20°C. *mfap-1(n4564 n5214); uaf-1(n4588 n5125)* mutants and *mfap-1(n4564 n5214); uaf-1(n4588 n5127)* mutants were viable at 15°C and became sterile at 20°C, and *mfap-1(n4564 n5214); uaf-1(n4588)* mutants were likely inviable at both 15°C and 20°C, since we failed to obtain progeny with this genotype from *mfap-1(n4564 n5214)/+; uaf-1(n4588)/+* hermaphrodites (Table 3). All viable *mfap-1; uaf-1* multiple mutant animals exhibited strong suppression of the *unc-93(e1500)* Unc phenotype, similar to that of the *mfap-1(n4564 n5214)* mutant animals (Table 3). That *mfap-1; uaf-1* multiple mutant animals, except *mfap-1(n4564 n5214)*; *uaf-1(n5123),* exhibited a stronger temperature-sensitive sterile or lethal phenotype than either *mfap-1* or *uaf-1* mutant animals suggests that the *mfap-1(n4564 n5214)* and *uaf-1* mutations might have additive effects on animal survival.

**Table 3:** Mutations in *mfap-1* and *uaf-1* interact to affect the locomotion of *unc-93(e1500)* animals.

Genotype	Body-bends per 30 sec ± SD	n
*wild-type* [29]	20.4±3.7	20
*unc-93(e1500)* [29]	0.9±1.2	20
*mfap-1(n5214); unc-93(e1500)*	1.8±1.3	20
*mfap-1(n4564 n5214); unc-93(e1500)*	21.8±4.5	20
*uaf-1(n5123) unc-93(e1500)* [29]	0.6±0.8	20
*mfap-1(n5214); uaf-1(n5123) unc-93(e1500)*	2.2±1.9	20
*mfap-1(n4564 n5214); uaf-1(n5123) unc-93(e1500)*	20.9±2.9	20
*uaf-1(n4588 n5125) unc-93(e1500)* [29]	5.4±2.1	20
*mfap-1(n5214); uaf-1(n4588 n5125) unc-93(e1500)*	10.2±5.7	20
*mfap-1(n4564 n5214); uaf-1(n4588 n5125) unc-93(e1500)* *	20.5±4.5	20
*uaf-1(n4588 n5127) unc-93(e1500)* [29]	21.5±3.6	20
*mfap-1(n5214); uaf-1(n4588 n5127) unc-93(e1500)*	21.2±4.4	20
*mfap-1(n4564 n5214); uaf-1(n4588 n5127) unc-93(e1500)* *	20.5±4.7	20
*uaf-1(n4588) unc-93(e1500)* [29]	21.0±5.5	20
*mfap-1(n5214); uaf-1(n4588) unc-93(e1500)* *	17.7±4.0	20
*mfap-1(n4564 n5214); uaf-1(n4588) unc-93(e1500)* **	Inviable	20
*mfap-1(n5214)*	28.4±3.3	20
*mfap-1(n4564 n5214)*	27.1±2.2	20
*uaf-1(n5123)* [29]	19.1±3.1	20
*uaf-1(n4588 n5125)* [29]	19.5±3.3	20
*uaf-1(n4588 n5127)* [29]	22.1±4.0	20
*uaf-1(n4588)* [29]	23.1±4.1	20

*: Sterile at 20°C.

**: Inviable.

We next examined whether *mfap-1(n5214)* could enhance the suppressor activities of *uaf-1* mutations for the Unc phenotype of *unc-93(e1500)* animals by generating *mfap-1(n5214); uaf-1* double mutant animals carrying the *unc-93(e1500)* mutation (Table 3). *mfap-1(n5214); uaf-1(n5123)* double mutants were similar to *mfap-1(n5214)* or *uaf-1(n5123)* single mutants and were not substantially suppressed for the Unc phenotype of *unc-93(e1500)* animals (Table 3). *mfap-1(n5214)* enhanced the suppressor activity of the *uaf-1(n4588 n5125)* mutation. As expected, both *mfap-1(n5214); uaf-1(n4588 n5127)* and *mfap-1(n5214); uaf-1(n4588)* strongly suppressed *unc-93(e1500)*, since both *uaf-1(n4588 n5127)* and *uaf-1(n4588)* are strong suppressors for *unc-93(e1500)* [29]. Although *mfap-1(n5214)* or *uaf-1(n4588 n5127)* mutants were apparently healthy at 20°C, *mfap-1(n5214); uaf-1(n4588 n5127)* mutant animals became partially sterile at 20°C (L. Ma and H. R. Horvitz, unpublished observations). Furthermore, *mfap-1(n5214); uaf-1(n4588)* double mutant animals were viable at 15°C but became sterile when grown at 20°C, which is a more severe temperature-sensitive phenotype than that of *uaf-1(n4588)* single mutants. That *mfap-1* mutations enhance the temperature-sensitive phenotypes of *uaf-1* mutants suggests that *mfap-1* and *uaf-1* might act in parallel in affecting *unc-93* function and animal survival.

We tested if *mfap-1(n4564 n5214),* like the *uaf-1(n4588)* mutation [29], would cause the recognition of a cryptic 3′ splice site in exon 9 of *unc-93(e1500)*. We did not observe any obviously altered recognition of this cryptic 3′ splice site (1.3% for wild type vs. 2% for *mfap-1(n4564 n5214)* animals) (Figure S1). Therefore, the suppression of the Unc phenotype of *unc-93(e1500)* by *mfap-1(n4564 n5214)* might not be caused by altered splicing of the *unc-93(e1500)* transcript.

### *mfap-1(n4564 n5214)* alters the splicing of *tos-1* by increasing intron 1 retention and exon 3 skipping

**Figure 3:**
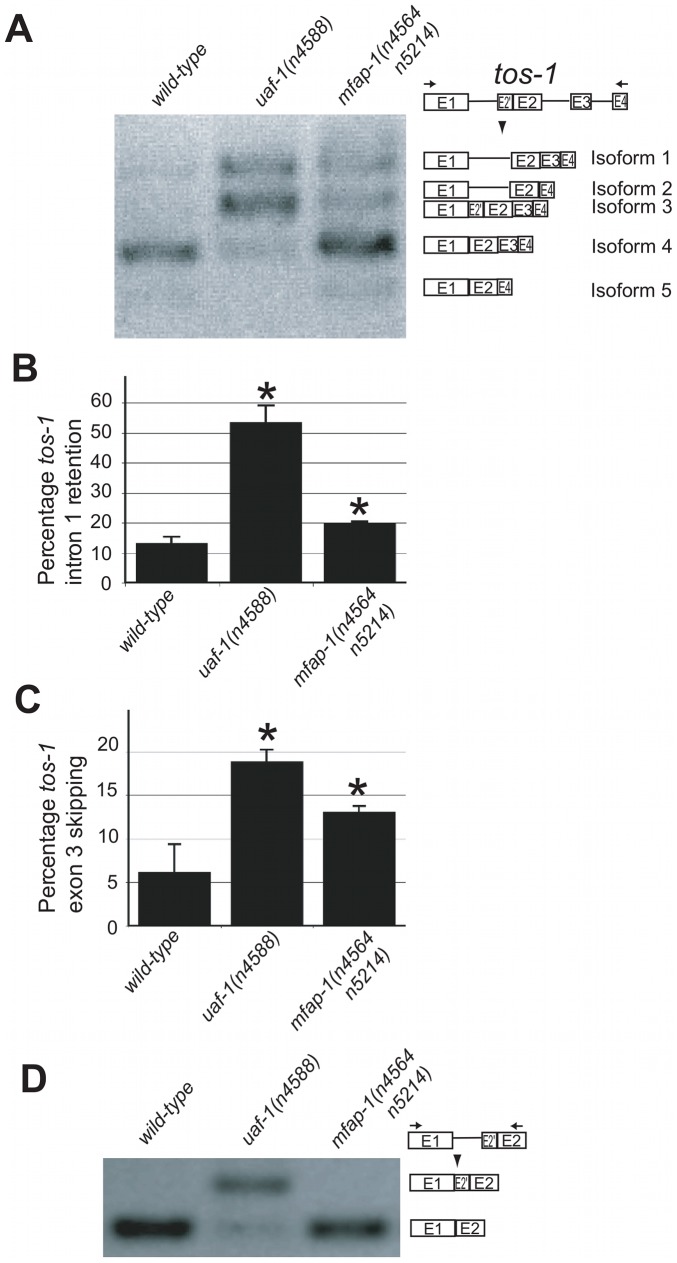
*mfap-1(n4564 n5214)* affects the splicing of *tos-1*. (A) RT-PCR experiments showing the effect of *mfap-1(n4564 n5214)* on *tos-1* alternative splicing. *tos-1* splice isoforms are illustrated on the right. (B) The molar ratios of all *tos-1* splice isoforms with intron 1 retention (isoforms 1 and 2), presented as a percentage of all isoforms combined (Isoforms 1, 2, 3, 4 and 5). Error bars: standard deviations. *p<0.05. (C) The molar ratios of all *tos-1* splice isoforms with exon 3 skipping (isoforms 2 and 5), presented as a percentage of all isoforms combined (isoforms 1, 2, 3, 4 and 5). Error bars: standard deviations. *p<0.05. (D) RT-PCR experiments showing the effect of the *mfap-1(n4564 n5214)* mutation on the recognition of the cryptic 3′ splice site of *tos-1* intron 1. For all analyses, isoform intensities were obtained by analyzing biological duplicates or triplicates using NIH ImageJ software.

To analyze whether *mfap-1* can affect alternative splicing, we examined the splicing of *tos-1* in *mfap-1(n4564 n5214)* animals. We previously described *tos-1* as a sensitive endogenous reporter for alternative splicing in *C. elegans* [30]. As shown in Figure 3A–3C, *mfap-1(n4564 n5214)* increased *tos-1* intron 1 retention and exon 3 skipping, suggesting that the recognition of the 3′ splice sites in intron 1 or before exon 3 was reduced in *mfap-1(n4564 n5214)* animals. We note that the *tos-1* splice isoforms we observed in *mfap-1(n4564 n5214)* and *mfap-1(RNAi)* animals can also be seen in wild-type animals (Figure 3A), indicating that alterations in *mfap-1* affect *tos-1* alternative splicing events that occur in the wild type. We also examined whether *mfap-1(n4564 n5214)* could cause recognition of the cryptic 3′ splice site in *tos-1* intron 1, which was recognized in *uaf-1(n4588)* animals [30]. We did not detect obvious recognition of the cryptic 3′ splice sites in *mfap-1(n4564 n5214)* animals (Figure 3D), suggesting that *mfap-1(n4564 n5214)* did not alter the specificity of 3′ splice-site recognition. We similarly examined *mfap-1(n5214)* animals and did not observe obvious effects on the splicing of *tos-1* or the recognition of the intron 1 cryptic 3′ splice site (Figure S2B), suggesting that *mfap-1(n5214)* does not alter or has a very weak effect on *tos-1* splicing. This result is consistent with the notion that *n5214* is a weaker *mfap-1* allele than *n4564 n5214*.

**Figure 4:**
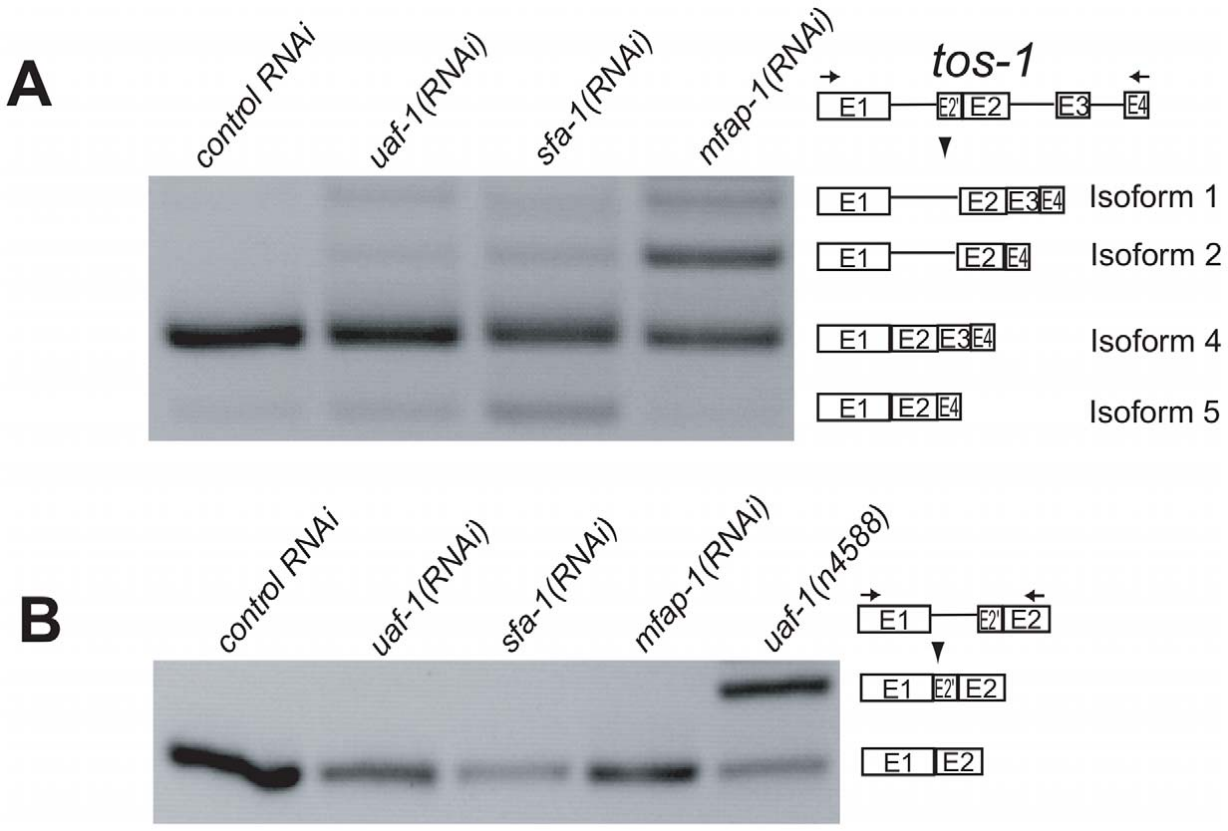
Reducing *mfap-1* expression by RNA interference alters the splicing of *tos-1*. (A) RT-PCR experiments showing the effect of reducing *mfap-1* expression by RNAi feeding on *tos-1* alternative splicing. *tos-1* splice isoforms are illustrated on right. (B) RT-PCR experiments showing the effect of reducing *mfap-1* expression by RNAi feeding on the recognition of the cryptic 3′ splice site of *tos-1* intron 1.

We next examined the splicing of *tos-1* in animals fed dsRNA targeting *mfap-1*. We observed an apparent increase of *tos-1* isoforms 1 and 2 (Figure 4A). Increased expression of isoforms 1 and 2 is caused by increased intron 1 retention and exon 3 skipping in *tos-1* splicing, as was seen for *mfap-1(n4564 n5214)* animals (Figure 3). The similarity of *mfap-1(n4564 n5214)* and *mfap-1(RNAi)* in affecting *tos-1* splicing further suggests that *n4564 n5214* causes a reduction or loss of *mfap-1* function. Reducing *mfap-1* expression by RNAi feeding did not cause the recognition of the cryptic 3′ splice site in intron 1 (Figure 4B). Similarly, that cryptic splice site was not recognized in *mfap-1(n4564 n5214)* animals (Figure 3D).

To determine whether *uaf-1* mutations and *mfap-1* mutations could interact in affecting RNA splicing, we examined the splicing of *tos-1* and the recognition of the cryptic 3′ splice site of *tos-1* intron 1 in *mfap-1; uaf-1* mutant animals. For each *mfap-1; uaf-1* mutant, the splicing of *tos-1* appeared to be similar to that in the corresponding *uaf-1* single mutant or *mfap-1* single mutant (Figure S2A, S2B, top panels). We did not observe additive or synergistic effects of *uaf-1* and *mfap-1* mutations on intron 1 retention (Figure S3A, *e.g.*, compare *uaf-1(n4588 n5127)* with *mfap-1(n4564 n5214); uaf-1(n4588 n5127)*) or exon 3 skipping (Figure S3B, *e.g.*, compare *mfap-1(n4564 n5214)* with *mfap-1(n4564 n5214); uaf-1(n4588 n5127)*). Interestingly, the recognition of the cryptic 3′ splice site was reduced by about 50% in *mfap-1(n4564 n5214); uaf-1(n4588 n5127)* and *mfap-1(n5214); uaf-1(n4588 n5127)* mutant animals compared to that in *uaf-1(n4588 n5127)* mutant animals (Figure S2A, S2B, bottom panels and Figure S3C), suggesting that wild-type *mfap-1* function might be required for the recognition of the cryptic 3′ splice site in *uaf-1(n4588 n5127)* animals.

### MFAP-1 interacts physically with known splicing factors in a yeast two-hybrid experiment

To further understand the function of MFAP-1, we sought to identify interacting proteins of MFAP-1 using a yeast two-hybrid screen (see Materials and Methods). We isolated and retested nine genes encoding proteins that might potentially interact with MFAP-1 (Table S1). Two of the genes, *K04G7.11* and *D1054.14*, encode the *C. elegans* orthologs of the presumptive splicing factors SYF2 [34] and Prp38 [35]. *Drosophila* MFAP-1 (dMFAP1) was found to be in a protein complex with dPrp38 in co-immunoprecipitation experiments and to interact with dPrp38 physically [33]. SYF2 mutations were found to cause synthetic lethality with mutations in splicing factors clf1Delta2 and prp17/cdc40Delta in *S. cerevisiae* [34], and mammalian orthologs of SYF2 were identified in various spliceosomal complexes by proteomic approaches [3], suggesting that SYF2 acts as a splicing factor. We also identified MFAP-1 as an interactor in the two-hybrid screen, indicating that MFAP-1 might form homodimers. The other potential interactors are two proteins involved in rRNA processing (C05C8.2 and T22H9.1), a ribosomal protein (RPS-6), a Ras-associated PH-domain containing protein (MIG-10), a Rab11 family-interacting protein 2 (Y39F10B.1) and a protein of unknown function (F43C11.9). Although the interactions between MFAP-1 and the proteins from the yeast two-hybrid screen remain to be verified by other approaches, the observation that two presumptive splicing factors (Prp38 and SYF2) are among the candidates is consistent with our findings and also those of Andersen and Tapon [33] indicating that MFAP-1 might function as a splicing factor.

We next examined the splicing of *tos-1* in animals fed dsRNAs targeting *mfap-1*, *D1054.14* and *K07G7.11* (Figure S4). We found that *D1054.14(RNAi)* caused an increase of *tos-1* splice isoform 2 similar to that seen in *mfap-1(RNAi)* animals (Figure S4). No apparent alteration in *tos-1* splicing was detected in *K07G7.11(RNAi)* animals. This result is consistent with previous studies indicating that homologs of D1054.14 can function as splicing factors [33], [35] and identified *tos-1* as a target of D1054.14 in *C. elegans*.

## Discussion

### MFAP-1 is essential for animal development

MFAP1 was initially identified as a putative extracellular matrix protein based on a screen of an embryonic chicken cDNA expression library using antibodies against elastic fiber microfibrils-enriched bovine ocular zonule proteins [32]. Our analysis of *C. elegans mfap-1* and the study of the *Drosophila* ortholog dMFAP1 [33] indicate that MFAP-1 orthologs in these two species are nuclear proteins that affect RNA splicing. In addition, the human ortholog of MFAP-1 was found to be associated with spliceosomes by mass spectrometry analysis [36]–[38]. Since the human ortholog of *mfap-1* can rescue the activity of *C. elegans mfap-1(n4564 n5214)* for suppressing the Unc phenotype of *unc-93(e1500)* animals, the function of MFAP-1 is likely conserved from nematodes to humans.

*Drosophila* dMFAP1 acts with several other splicing factors in G2/M progression during mitosis and affects the ratio of pre-mRNA to mature mRNA of the γ*-tubulin* gene and the mRNA level of the *stg/cdc25* gene [33]. It is not known if dMFAP1 affects alternative splicing [33]. *C. elegans mfap-1(n4564 n5214)* mutants exhibit a temperature-sensitive phenotype with normal growth at 15°C and embryonic lethality at 25°C; *mfap-1(tm3456*Δ) homozygous animals arrest at L1 to L2 larval stages at 20°C, and *mfap-1* RNAi-treated animals arrest at variable larval stages at 20°C (L. Ma and H. R. Horvitz, unpublished observations). These observations indicate that *C. elegans mfap-1* is essential for animal development. Whether the developmental defect of *mfap-1-*deficient animals is caused by mitotic defects remains to be determined.

### MFAP-1 is probably required for the recognition of weak 3′ splice sites

We isolated *mfap-1(n4564 n5214)* from the same genetic screen in which we identified mutations affecting *uaf-1* and *sfa-1* [29]. *mfap-1(n4564 n5214)* suppressed the phenotypes of different rubberband Unc mutants in patterns similar to those of *uaf-1(n4588)* and *sfa-1(n4562)* mutations. The findings led us to hypothesize that MFAP-1 might function as a splicing factor. Our analysis of *tos-1* splicing in *mfap-1* mutant animals and in animals treated with *mfap-1* RNAi provided molecular evidence that *mfap-1* can affect alternative splicing. *mfap-1(n4564 n5214)* and *mfap-1(RNAi)* both increased *tos-1* intron 1 retention (with a weak 3′ splice site) and *tos-1* exon 3 skipping (with a different weak 3′ splice site), and neither affected the splicing of intron 3 (with a strong consensus 3′ splice site TTTTCAG). These observations suggest that MFAP-1 might be required for the recognition of weak 3′ splice sites and not for the recognition of strong 3′ splice sites. We propose that MFAP-1 can act much like UAF-1 and SFA-1, mutations in which cause reduced recognition of the weak 3′ splice sites in introns 1 and 2 and do not affect the recognition of the consensus 3′ splice site in intron 3 of *tos-1* [30]. Similar to *sfa-1(n4562)*, *mfap-1(n4564 n5214)* or *mfap-1(RNAi)* did not cause recognition of the cryptic 3′ splice site in *tos-1* intron 1, suggesting that MFAP-1 probably is not essential for determining the specificity of 3′ splice sites.

The effects of *mfap-1(n4564 n5214)* and *mfap-1(RNAi)* on *tos-1* splicing are qualitatively similar but quantitatively different. We also observed differences in the effects on *tos-1* splicing by genetic mutations and RNAi treatments in our previous studies of *uaf-1* and *sfa-1*, in which we found that *uaf-1(n4588)* or *sfa-1(n4562)* caused more dramatic alterations of *tos-1* splicing than did either *uaf-1(RNAi)* or *sfa-1(RNAi)*. This difference was at least partially caused by an altered function of UAF-1 in *uaf-1(n4588)* animals [30]. Thus, differing effects on target gene splicing by RNAi-treatment and genetic mutation of a splicing factor gene could be caused by several factors, including the differing level of the splicing factor in RNAi-treated and mutant animals and the effects of mutations on the function of the splicing factor.

### MFAP-1 might interact with multiple partners to affect RNA splicing

From a yeast two-hybrid screen, we identified nine candidate MFAP-1-interacting proteins. One, D1054.14, is homologous to the *S. cerevisiae* splicing factor Prp38 [35]. Another, K04G7.11, is orthologous to the *S. cerevisiae* candidate splicing factor SYF2 [34]. *Drosophila* splicing factors dPrp38 and dSYF1 were found in a protein complex with dMFAP1, and dPrp38 interacts with dMFAP1 in GST-pulldown experiments [33]. We did not identify orthologs of SYF1 or several other splicing factors found in the *Drosophila* dMFAP1 complex, possibly because the co-immunoprecipitation experiments could have identified *Drosophila* proteins that did not directly interact with dMFAP1, whereas our yeast two-hybrid screen could identify only proteins that directly interact with MFAP-1. Both studies suggest that MFAP-1 interacts with multiple splicing factors directly or indirectly and hence that MFAP-1 might play multiple roles in affecting RNA splicing.

We found that reducing the expression of *D1054.14* by RNAi caused alterations in *tos-1* splicing similar to those caused by *mfap-1(RNAi)* (Figure S4), suggesting that MFAP-1 and D1054.14/PRP38 might interact to regulate the splicing of the same set of genes. We did not identify alterations in *tos-1* splicing in animals fed dsRNA targeting *K04G7.11*, indicating that *K04G7.11* might not be required for the splicing of *tos-1*.

### *mfap-1* and *uaf-1* might interact to affect RNA splicing

We previously concluded that the suppression of the rubberband Unc phenotype of *unc-93(e1500)* animals by *uaf-1(n4588)* and *sfa-1(n4562)* is probably caused by altered splicing of an unknown gene [29]. The splicing of *unc-93(e1500)* appears to be normal in *mfap-1(n4564 n5214)* animals, indicating that altered splicing of *unc-93(e1500)* transcripts similarly is not the cause of the suppression of *unc-93(e1500)* Unc phenotype by *mfap-1(n4564 n5214)*.

The *uaf-1(n4588)* mutation likely causes the recognition of the cryptic 3′ splice site of *tos-1* intron 1 by altering rather than decreasing the function of the UAF-1 protein [30]. *uaf-1(n4588 n5127)* is a weaker mutation than *uaf-1(n4588)*, and the recognition of the cryptic 3′ splice site in *uaf-1(n4588 n5127)* animals but not in *uaf-1(n4588)* animals was reduced by the presence of the *mfap-1(n4564 n5214)* and *mfap-1(n5214)* mutations (Figure S3C). These results suggest that the recognition of the cryptic 3′ splice site of *tos-1* requires MFAP-1 only if that recognition is already weak as in *uaf-1(n4588 n5127)* mutants. These results further support our conclusion that MFAP-1 affects alternative splicing.

In short, we propose that MFAP-1 can act as a splicing factor. Future studies should reveal how MFAP-1 interacts with other splicing factors to affect *C. elegans* development by regulating splicing of its target genes.

## Materials and Methods

### Strains

*C. elegans* strains were grown at 20°C as described unless otherwise indicated [39]. N2 (Bristol) was the reference wild-type strain [39]. Other strains used in the study were:

LGI: *mfap-1(n4564 n5214, n5214, tm3456*Δ) (this study).

LGII: *sup-9(n1550)* [20].

LGIII: *uaf-1(n4588, n5123, n4588 n5125, n4588 n5127)* [29], *unc-93(e1500, n200)* [23], *sup-18(n1014)* [21].

LGIV: *sfa-1(n4562)* [29].

LGX: *sup-10(n983)* [21].

CB4856 (Hawaiian) [40]

We used the genetic translocation *hT2[bli-4(e937) let-?(q782) qIs48]* LG I; LG III [41], which carries an integrated pharyngeal GFP element [42], to balance the *mfap-1(tm3456*Δ) mutation.

### Cloning of *mfap-1*

We used single-nucleotide polymorphism (SNP) mapping [40] to localize *n4564 n5214* to the right of nucleotide 7110 (SNP T01H8: 7110 S = AT) on cosmid T01H8 (T01H8 sequences refer to nucleotides of GenBank accession no. Z80219) and to the left of nucleotide 3334 (SNP F36A2: 3334 S = CT) on cosmid F36A2 (F36A2 sequences refer to nucleotides of GenBank accession no. Z81077) (Figure 1A). We isolated 10 Dpy recombinants after crossing *dpy-5(e61) n4564 n5214* hermaphrodites with males of the Hawaiian strain CB4856, and six Unc recombinants after crossing *n4564 n5214 unc-75(e950)* hermaphrodites with CB4856 males. Cosmid *F43G9* rescued three phenotypic characteristics of the *n4564 n5214* strain: suppression of *unc-93(e1500)*, partial sterility and temperature-sensitive lethality at 25°C (Figure 1B). We determined the coding sequences of *F43G9.10* and identified two missense mutations (GAT-to-GTT (D426V) and ACA-to-GCA (T427A)) in the fourth exon of *F43G9.10* (Figure 1C). The predicted proteins encoded by the *F43G9.10* orthologs in *C. elegans*, *Drosophila*, chicken and human are highly conserved, and the Asp-Thr dipeptide altered in the *mfap-1(n4564 n5214)* mutant is completely conserved (Figure 1D). A genomic fragment containing a 2.5 kb promoter region, the entire 1.7 kb coding region and a 1.5 kb 3′ downstream region exhibited rescuing activity similar to that of the *F43G9* cosmid (Figure 1B). Transgenes expressing *F43G9.10* and or the human ortholog of *F43G9.10* (*Hsmfap-1*) in body-wall muscles each rescued the suppression of *unc-93(e1500)* by *mfap-1(n4564 n5214)* (Figure 1B). A transgene carrying the *mfap-1(n4564 n5214)* mutation (*mut*) failed to rescue the suppression of *unc-93(e1500)* by *mfap-1(n4564 n5214)* (Figure 1B), suggesting that this mutation caused the suppressor activity in *mfap-1(n4564 n5214)* animals.

### Screen for suppressors of *mfap-1(n4564 n5214)*

Synchronized L4 *mfap-1(n4564 n5214)* animals (P_0_) grown at 15°C were mutagenized with ethyl methanesulfonate (EMS) as described [39] and grown to gravid adults at 15°C. The P_0_ adults were bleached, and their F_1_ progeny were grown to young adults at 15°C and moved to 25°C. After three days at 25°C, the plates were examined for surviving fertile animals. From about 50,000 haploid genomes (F_2_) screened, two independent suppressed strains were isolated. As animals with a *mfap-1(n4564 n5214)* phenotype (partial sterility at 20°C and temperature-sensitive lethality at 25°C) were not identified from about 1800 progeny of *mfap-1(n4564 n5214) sup/++*animals, we concluded that the two suppressors were either intragenic or extragenic and very closely linked to *mfap-1*. DNA sequence determination identified in both suppressors the same nucleotide change (GCA-to-ACA at codon 427), which converted the mutated amino acid alanine in the *mfap-1(n4564 n5214)* mutant to the wild-type threonine (A427T) while retaining the D426V mutation. One of the two isolates was used for subsequent studies, and the single mutation it carried was designated *mfap-1(n5214)*.

### Yeast two-hybrid screen

We used a yeast two-hybrid screen [43], [44] to identify proteins that might interact with MFAP-1. A pACT2.2 *C. elegans* yeast two-hybrid library (Addgene plasmid 11523, provided by Dr. Guy Caldwell) was used to screen for MFAP-1 interactors. For the bait, a full-length *mfap-1* cDNA was subcloned into the pGBK T7 plasmid (Clontech) and then transfected into the *S. cerevisiae* strain PJ69-4A. pGBK T7-*mfap-1*-positive yeast cells were transfected with 2 µg of DNA from the pACT2.2 yeast two-hybrid library, and cells were grown on a SD-Leu-Trp-His medium with 5 mM 3-AT. Positive clones were picked and grown in separate cultures using the same SD medium. Plasmids were obtained from each clone by the smash-and-grab method [45]. Purified plasmids were transfected into the bacterial strain DH5α and grown on LB-ampicillin plates. Plasmids were purified from the bacterial cultures, and sequences of the inserts were determined. Using yeast two-hybrid assays, we confirmed that these clones did not cause survival of yeast cells transfected with the pGBK T7 empty vector on SD-Leu-Trp-His medium, suggesting that proteins encoded by these clones potentially interact with MFAP-1 but not with the GAL4 DBD domain expressed by the pGBK T7 vector.

### RNA interference

Young adult animals were fed HT115 (DE3) bacteria containing plasmids directing the expression of dsRNAs targeting *uaf-1, sfa-1*, *mfap-1, D1054.14* or *K04G7.11* on NGM plates with 1 mM IPTG and 0.1 mg/ml Ampicillin [46]. F_1_ progeny of these animals, which arrested at various developmental stages, were washed from plates, rinsed with H_2_O and resuspended in Trizol (Invitrogen) for preparation of total RNA. The DNA construct that expresses dsRNA targeting *uaf-1* and the bacterial strain that expresses dsRNA targeting *sfa-1* were described previously [29]. We obtained a bacterial strain expressing dsRNA targeting *mfap-1* from a whole-genome RNAi library [47] and bacterial strains expressing dsRNAs targeting *mfap-1*, *D1054.14* or *K04G7.11* from an ORFeome-based RNAi library [48]. The sequences of plasmids from single colonies were determined to confirm the presence of coding sequences for each gene.

### Body-bend assay

L4 animals were picked 16–24 hrs before being tested. One day later, young adults were individually picked to plates with OP50 bacteria, and body-bends were counted for 30 sec using a dissecting microscope as described [49].

### Molecular biology

Total RNA was purified with Trizol (Invitrogen), and cDNA was generated following the protocol provided with the Superscript II kit (Invitrogen). PCR was performed with Eppendorf Cyclers, and DNA products were resolved using agarose gels. NIH ImageJ software was used to quantify *tos-1* splice isoforms [30]. The percentages of *tos-1* intron 1 retention, exon 3 skipping and the recognition of the intron 1 cryptic 3′ splice were analyzed as described [30]. We performed RT-PCR experiments with animals at different developmental stages and found no indication that *tos-1* splicing is regulated developmentally (Figure S5). DNA sequence analysis was performed using an ABI Prism 3100 Genetic Analyzer and an ABI 3730XL DNA Analyzer.

### Plasmids

*mfap-1* cDNAs were amplified by RT-PCR from wild-type, *mfap-1(n4564 n5214)* or *mfap-1(n5214)* animals and subcloned to the pPD93.97 vector in-frame with GFP using *BamH*I blunt/*Age*I restriction sites. For constructs expressing the *mfap-1(n4564)* transgene, the *n4564* mutation was introduced into the pPD93.97::*mfap-1cDNA(wt)* construct using a QuickChange Site-Directed Mutagenesis Kit (Stratagene) with primers containing the *n4564* mutation. For the plasmid used as bait in the yeast two-hybrid screen, full-length *mfap-1* cDNA was released from the pGEM-T Easy vector (Promega) with *Nco*I blunt/*Pst*I and subcloned in-frame into the pGBK T7 vector (Trp1, Kan^r^) (Clontech) using *BamH*I blunt/*Pst*I restriction sites. To construct a P*_mfap-1_::GFP* transcriptional fusion transgene, an *mfap-1* promoter fragment of about 2.5 kb was amplified using PCR and subcloned into the pPD95.79 vector using *Pst*I/*Sma*I restriction sites.

### Transgene experiments

Microinjection of DNA into the syncytial gonad and the generation of animals with germline transmission of transgenes were performed as described [50] with *mfap-1(n4564 n5214)* animals grown at 15°C. DNA injection mixtures generally contained 20 µg/ml 1 kb DNA ladder, 20 µg/ml *Arabidopsis* genomic DNA and 20 µg/ml of the transgene of interest. When the transgene did not express a GFP fusion protein, 20 µg/ml pPD95.86-GFP plasmid (which expresses GFP in body-wall muscles) was added to the injection mixture as a visible fluorescence marker.

## Supporting Information

Figure S1 *mfap-1(n4564 n5214)* does not obviously affect the altered splicing of *unc-93(e1500)* exon 9. Real-time RT-PCR was performed as described [29] to quantify the recognition of the cryptic 3′ splice site of *unc-93(e1500)* exon 9. No apparent difference between *unc-93(e1500)* and *mfap-1(n4564 n5214); unc-93(e1500)* animals was detected. *: results reported previously by Ma and Horvitz (2009) [29].
(TIF)

Figure S2 *mfap-1* and *uaf-1* mutations do not exhibit additive or synergistic effects on the altered splicing of *tos-1*. (A) RT-PCR experiments showing the splicing of *tos-1* (top panel) or the recognition of the cryptic 3′ splice site of *tos-1* intron 1 (bottom panel) in *mfap-1(n4564 n5214)* single or *mfap-1(n4564 n5214); uaf-1* mutant animals. Genotypes are indicated at the top, and *tos-1* splice isoforms on the right. (B) RT-PCR experiments showing the splicing of *tos-1* (top panel) or the recognition of the cryptic 3′ splice site of *tos-1* intron 1 (bottom panel) in *mfap-1(n5214)* single or *mfap-1(n5214); uaf-1* double mutant animals. Genotypes are indicated at the top, and *tos-1* splice isoforms on the right.
(TIF)

Figure S3 Quantification of *tos-1* intron 1 retention, exon 3 skipping and the recognition of the intron 1 cryptic 3′ splice site in *mfap-1; uaf-1* multiple mutant animals. (A) The molar ratios of all *tos-1* splice isoforms with intron 1 retention, presented as a percentage of all isoforms combined. Error bars: standard deviations. (B) The molar ratios of all *tos-1* splice isoforms with exon 3 skipping, presented as a percentage of all isoforms combined. Error bars: standard deviations. (C) Percentages of *tos-1* isoforms spliced at the cryptic 3′ splice site of *tos-1* intron 1 compared to all isoforms spliced at either the endogenous 3′ splice site or the cryptic 3′ splice site. For all analyses, isoform intensities were obtained by analyzing biological duplicates or triplicates using NIH ImageJ software. N.S., no significant difference.
(TIF)

Figure S4 Reducing the expression of *mfap-1* or *D1054.14* by RNA interference altered the splicing of *tos-1*. RT-PCR experiments showing the effects of reducing the expression of *mfap-1*, *D1054.14* or *K04G7.11* by RNAi feeding on *tos-1* alternative splicing. *mfap-1(RNAi)* and *D1054.14(RNAi)* caused similar alterations in *tos-1* splicing, while *K04G7.11(RNAi)* did not obviously affect the splicing of *tos-1*. RNAi bacterial strains were obtained from an ORFeome-based RNAi library [48].
(TIF)

Figure S5 The splicing of *tos-1* is not regulated developmentally. RT-PCR experiments examining the splicing of *tos-1* in animals at different synchronized developmental stages. *tos-1* splicing was similar in all developmental stages examined. YA: young adult animals 24 hours after the L4 larval stage.
(TIF)

Table S1 Candidate MFAP-1 interactors isolated from a yeast two-hybrid screen.
(DOC)
