# Supplementary figures and images for: The Caenorhabditis elegans Gene mfap-1 Encodes a Nuclear Protein That Affects Alternative Splicing
Source: PLoS Genet. 2012 Jul 19;8(7):e1002827. doi: 10.1371/journal.pgen.1002827 (PMC3400559; doi:10.1371/journal.pgen.1002827)

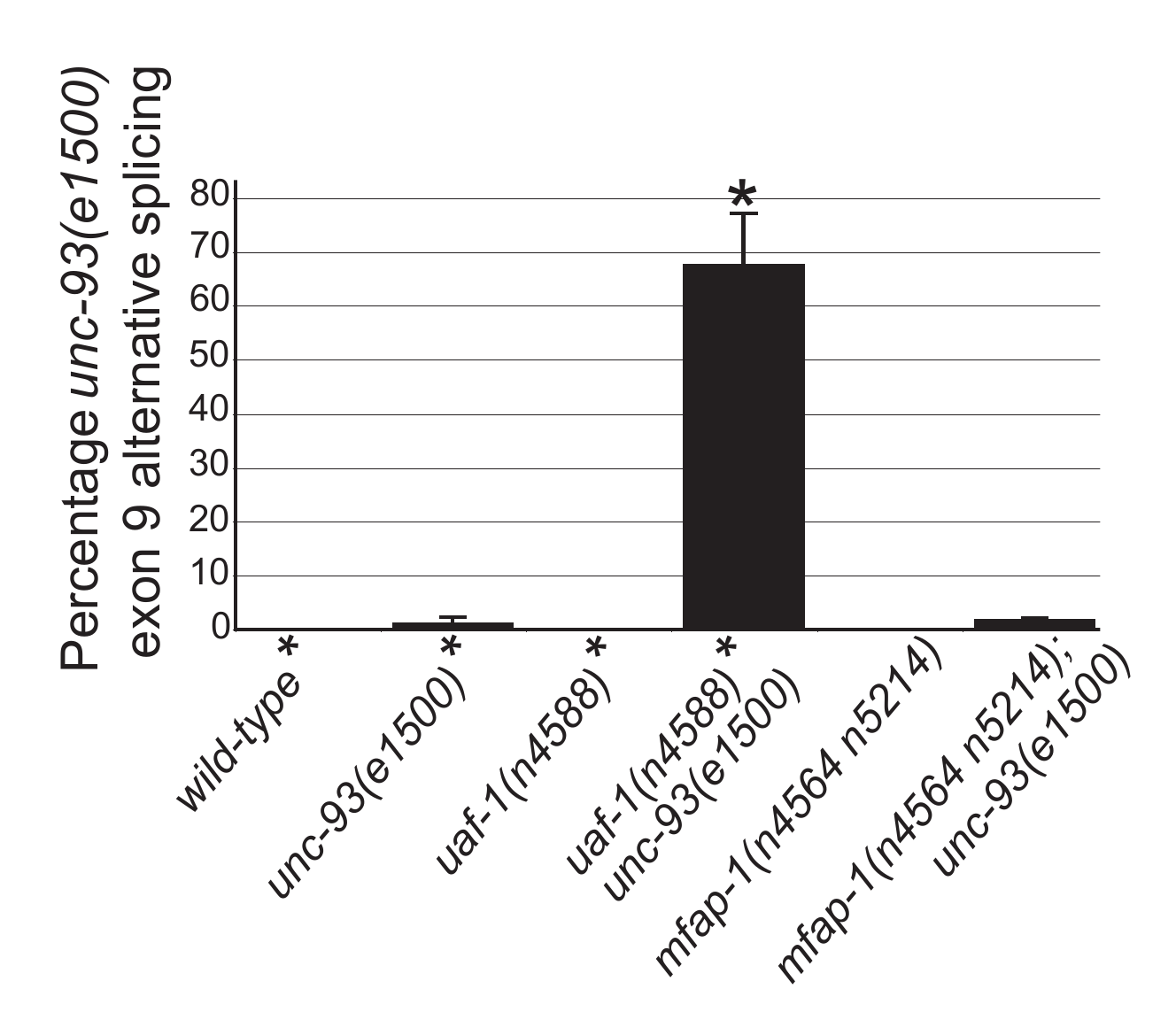

Supplement: Figure S1 — mfap-1(n4564 n5214) does not obviously affect the altered splicing of unc-93(e1500) exon 9. Real-time RT-PCR was performed as described [29] to quantify the recognition of the cryptic 3′ splice site of unc-93(e1500) exon 9. No apparent difference between unc-93(e1500) and mfap-1(n4564 n5214); unc-93(e1500) animals was detected. *: results reported previously by Ma and Horvitz (2009) [29]. (TIF) [file pgen.1002827.s001.tif]

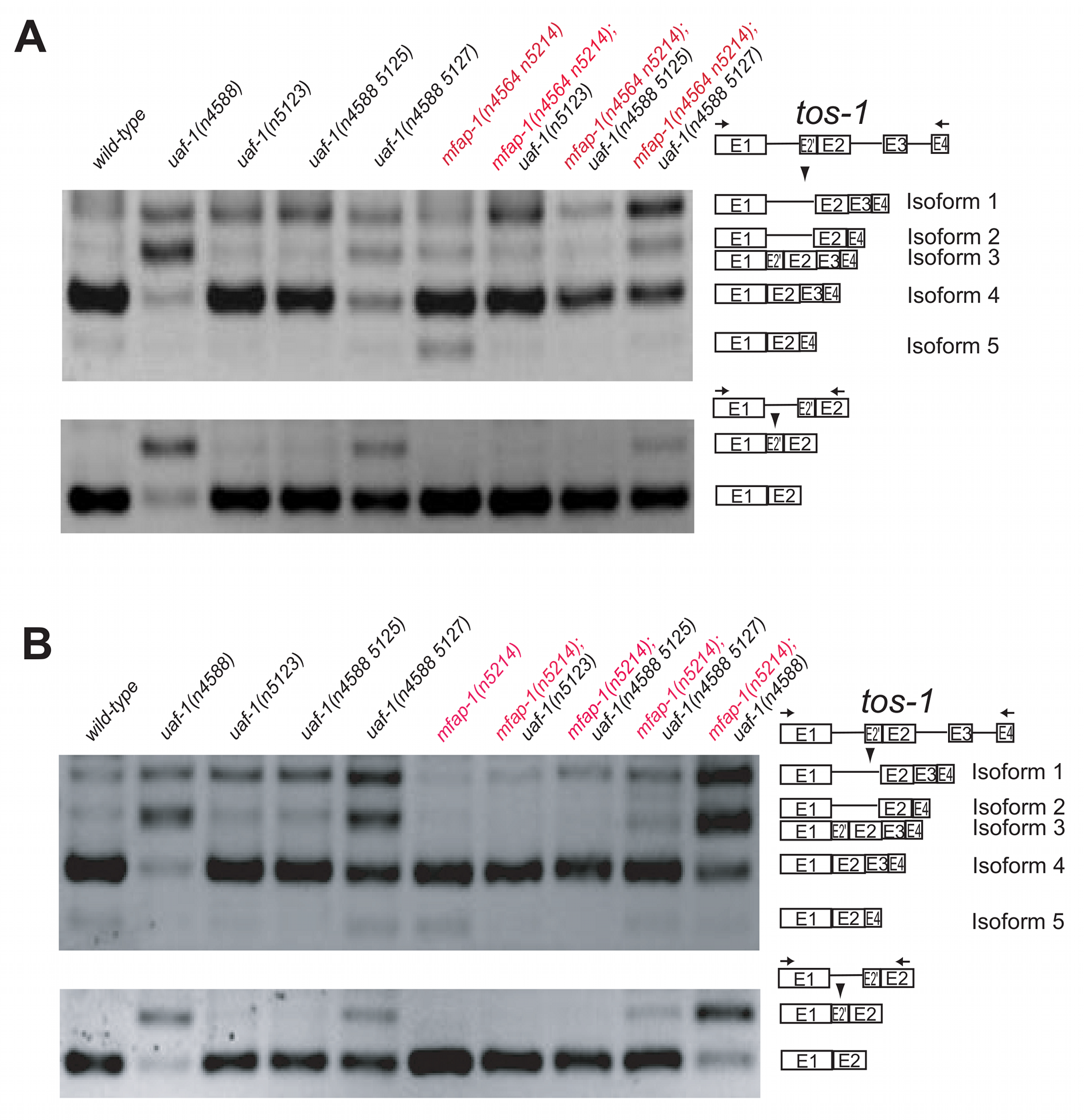

Supplement: Figure S2 — mfap-1 and uaf-1 mutations do not exhibit additive or synergistic effects on the altered splicing of tos-1. (A) RT-PCR experiments showing the splicing of tos-1 (top panel) or the recognition of the cryptic 3′ splice site of tos-1 intron 1 (bottom panel) in mfap-1(n4564 n5214) single or mfap-1(n4564 n5214); uaf-1 mutant animals. Genotypes are indicated at the top, and tos-1 splice isoforms on the right. (B) RT-PCR experiments showing the splicing of tos-1 (top panel) or the recognition of the cryptic 3′ splice site of tos-1 intron 1 (bottom panel) in mfap-1(n5214) single or mfap-1(n5214); uaf-1 double mutant animals. Genotypes are indicated at the top, and tos-1 splice isoforms on the right. (TIF) [file pgen.1002827.s002.tif]

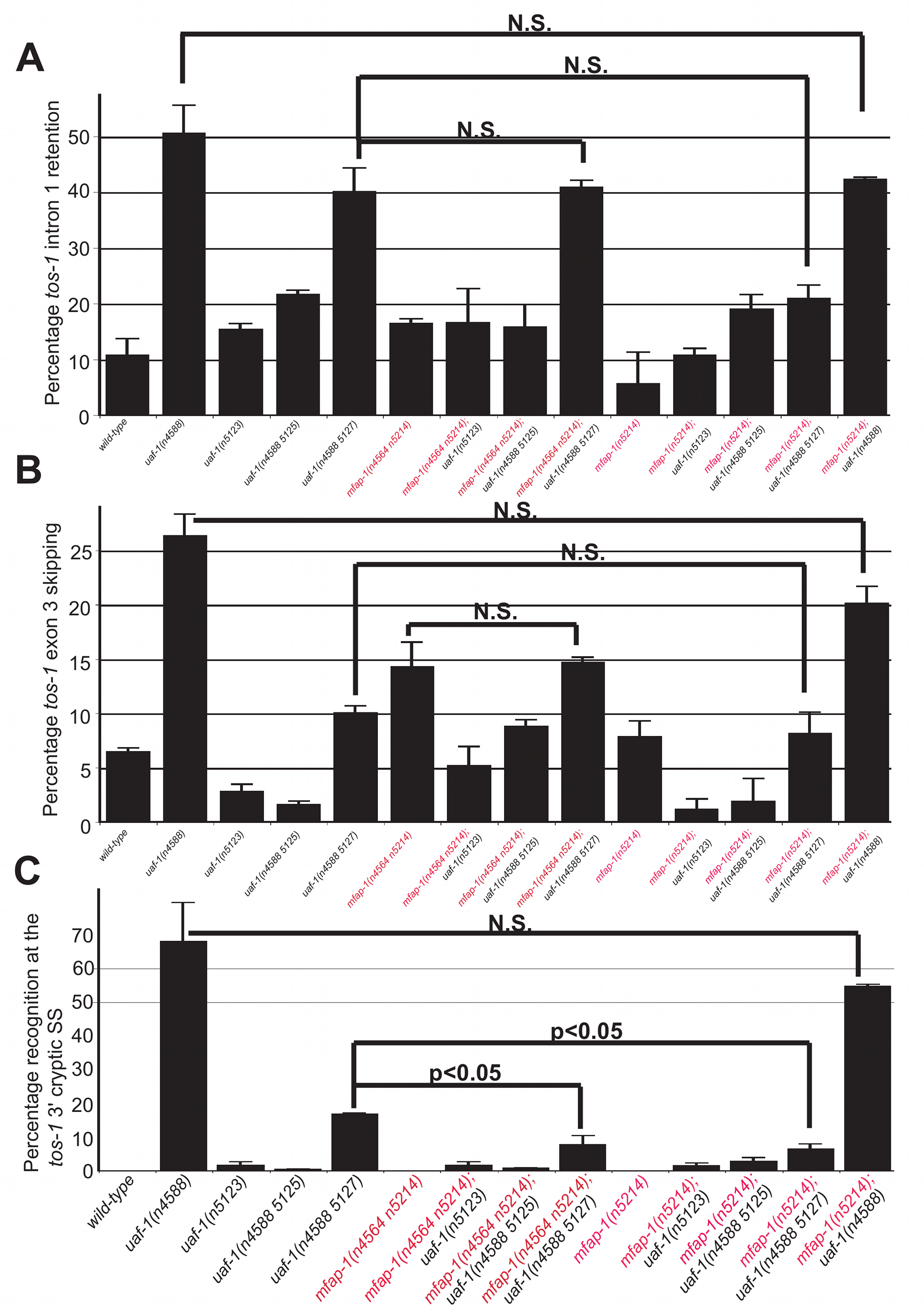

Supplement: Figure S3 — Quantification of tos-1 intron 1 retention, exon 3 skipping and the recognition of the intron 1 cryptic 3′ splice site in mfap-1; uaf-1 multiple mutant animals. (A) The molar ratios of all tos-1 splice isoforms with intron 1 retention, presented as a percentage of all isoforms combined. Error bars: standard deviations. (B) The molar ratios of all tos-1 splice isoforms with exon 3 skipping, presented as a percentage of all isoforms combined. Error bars: standard deviations. (C) Percentages of tos-1 isoforms spliced at the cryptic 3′ splice site of tos-1 intron 1 compared to all isoforms spliced at either the endogenous 3′ splice site or the cryptic 3′ splice site. For all analyses, isoform intensities were obtained by analyzing biological duplicates or triplicates using NIH ImageJ software. N.S., no significant difference. (TIF) [file pgen.1002827.s003.tif]

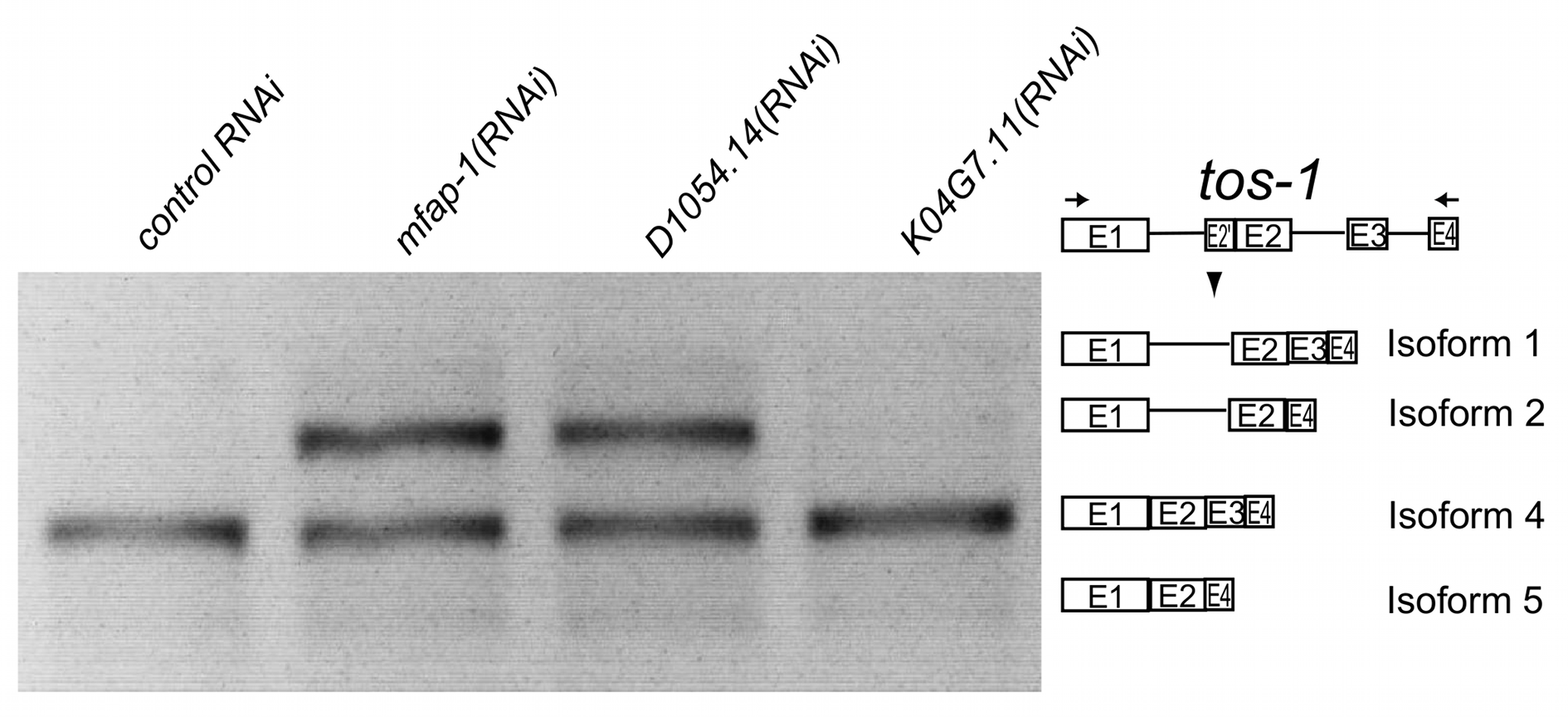

Supplement: Figure S4 — Reducing the expression of mfap-1 or D1054.14 by RNA interference altered the splicing of tos-1. RT-PCR experiments showing the effects of reducing the expression of mfap-1, D1054.14 or K04G7.11 by RNAi feeding on tos-1 alternative splicing. mfap-1(RNAi) and D1054.14(RNAi) caused similar alterations in tos-1 splicing, while K04G7.11(RNAi) did not obviously affect the splicing of tos-1. RNAi bacterial strains were obtained from an ORFeome-based RNAi library [48]. (TIF) [file pgen.1002827.s004.tif]

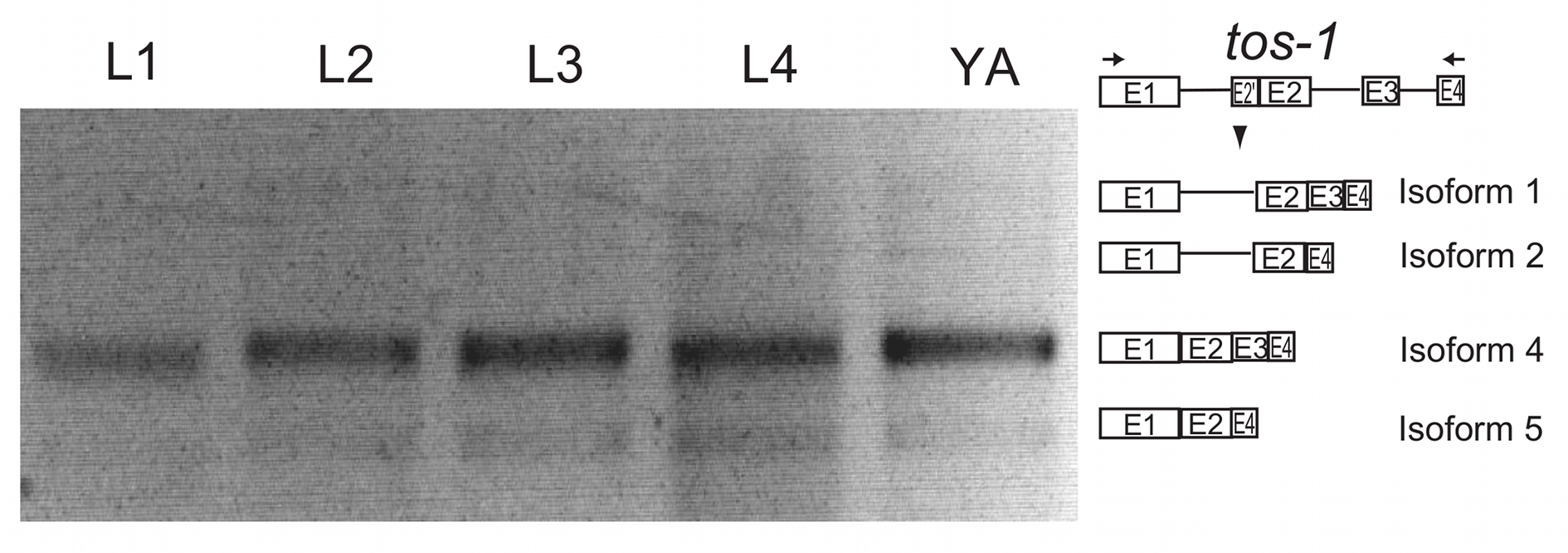

Supplement: Figure S5 — The splicing of tos-1 is not regulated developmentally. RT-PCR experiments examining the splicing of tos-1 in animals at different synchronized developmental stages. tos-1 splicing was similar in all developmental stages examined. YA: young adult animals 24 hours after the L4 larval stage. (TIF) [file pgen.1002827.s005.tif]
