# Supplementary material for: The Caenorhabditis elegans Gene mfap-1 Encodes a Nuclear Protein That Affects Alternative Splicing
Source: PLoS Genet. 2012 Jul 19;8(7):e1002827. doi: 10.1371/journal.pgen.1002827 (PMC3400559; doi:10.1371/journal.pgen.1002827)
Supplement: Table S1 — Candidate MFAP-1 interactors isolated from a yeast two-hybrid screen. (DOC) [file pgen.1002827.s006.doc]

| **Gene** | **Annotated Protein Function** |
| --- | --- |
|  |  |
| *D1054.14* | Splicing factor Prp38 |
| *K04G7.11* | Splicing factor SYF2 |
| *mfap-1* | MFAP-1 |
| *C05C8.2* | rRNA processing protein |
| *T22H9.1* | rRNA processing protein |
| *rps-6* | Small ribosome subunit protein |
| *mig-10* | Ras-association, a PH domain |
| *Y39F10B.1* | Rab11 family-interacting protein 2 |
| *F43C11.9* | Novel |
